# Supplementary figures and images for: Using whole-brain diffusion tensor analysis to evaluate white matter structural correlates of delayed visuospatial memory and one-week motor skill retention in nondemented older adults: A preliminary study
Source: PLoS One. 2022 Sep 22;17(9):e0274955. doi: 10.1371/journal.pone.0274955 (PMC9499308; doi:10.1371/journal.pone.0274955)

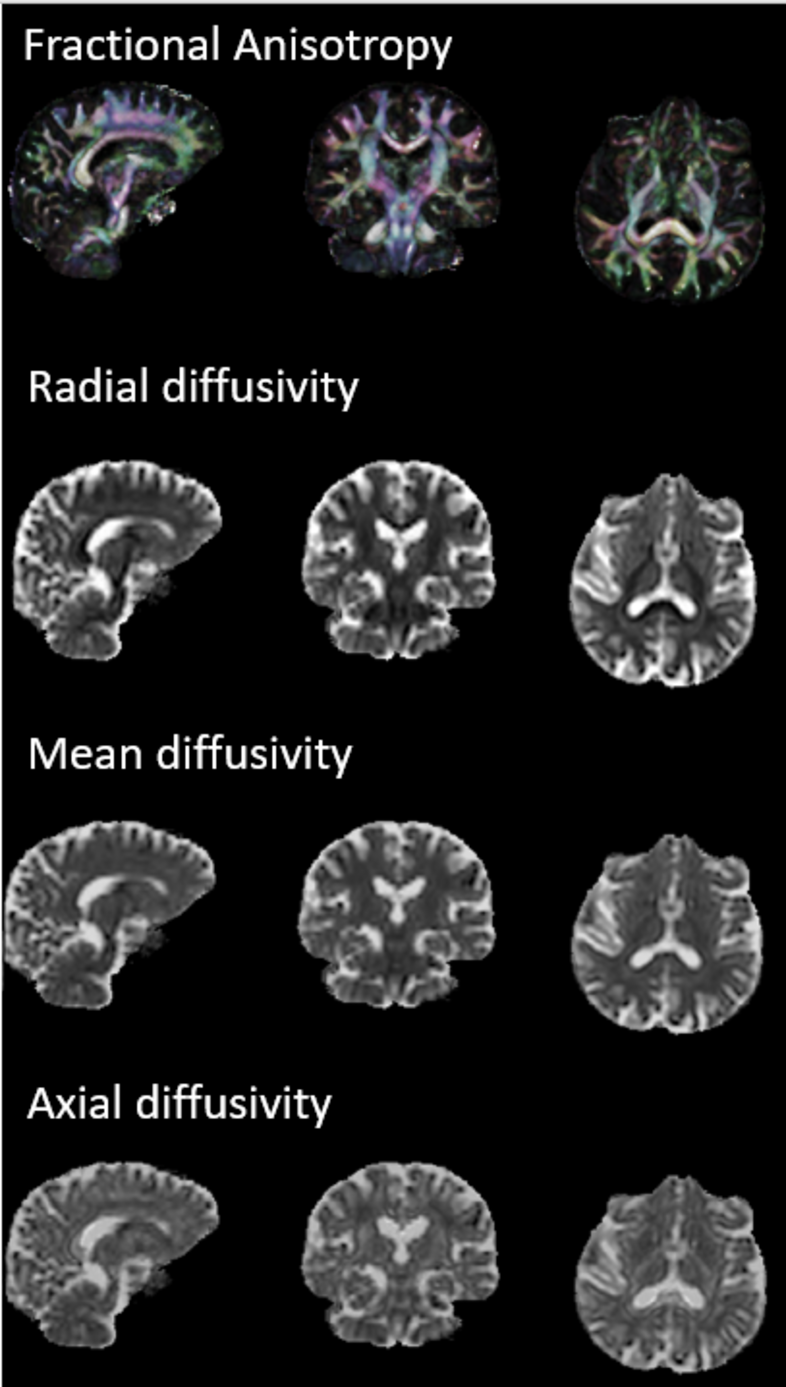

Supplement: S1 Fig — Fractional anisotropy (top row), radial (second row), mean (third row), and axial (bottom row) diffusivity maps for an example participant that demonstrates the diffusion tensor model fit the data as expected. (TIF) [file pone.0274955.s001.tif]

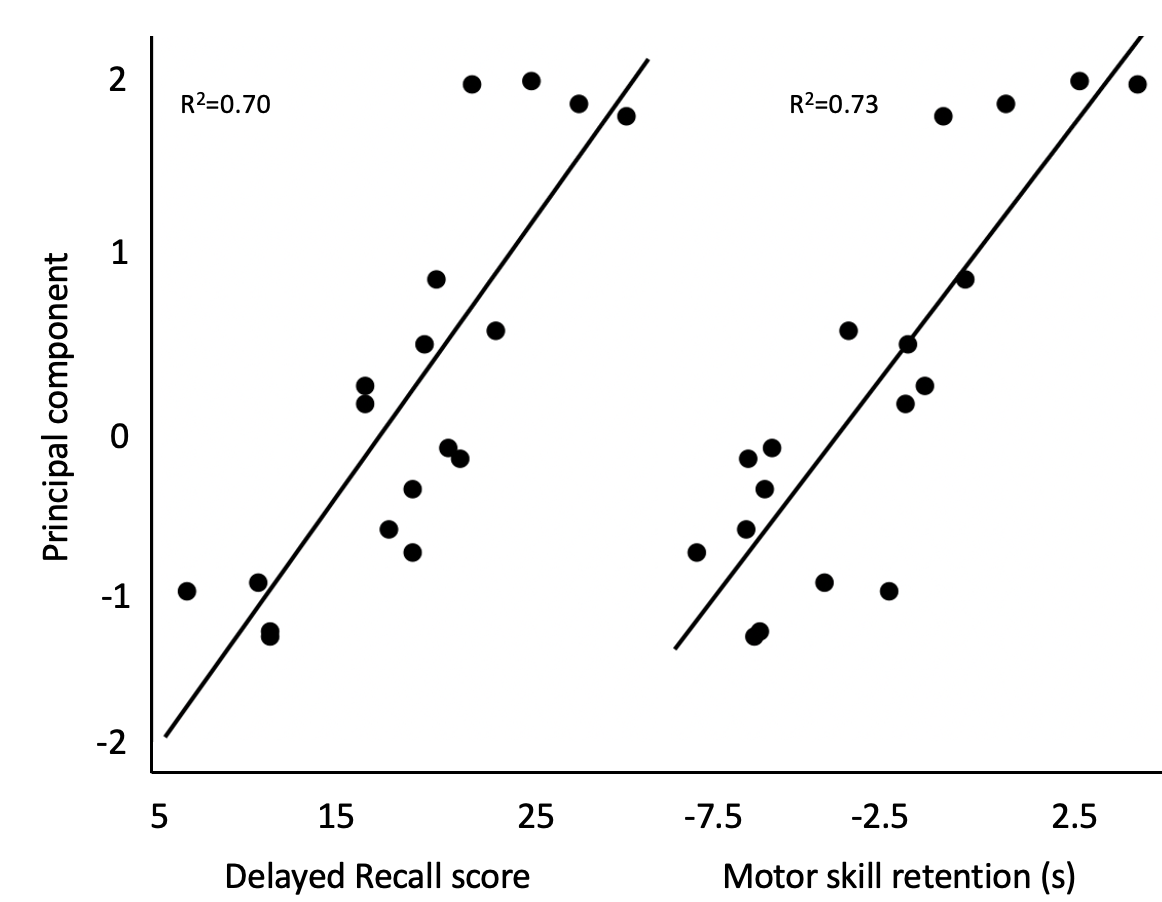

Supplement: S2 Fig — Principal component values (y-axis) for each participant was highly correlated with their one-week skill retention and Delayed Recall scores (x-axes), demonstrating that it was indeed a good representation of the shared variance of both behaviors. (TIF) [file pone.0274955.s002.tif]

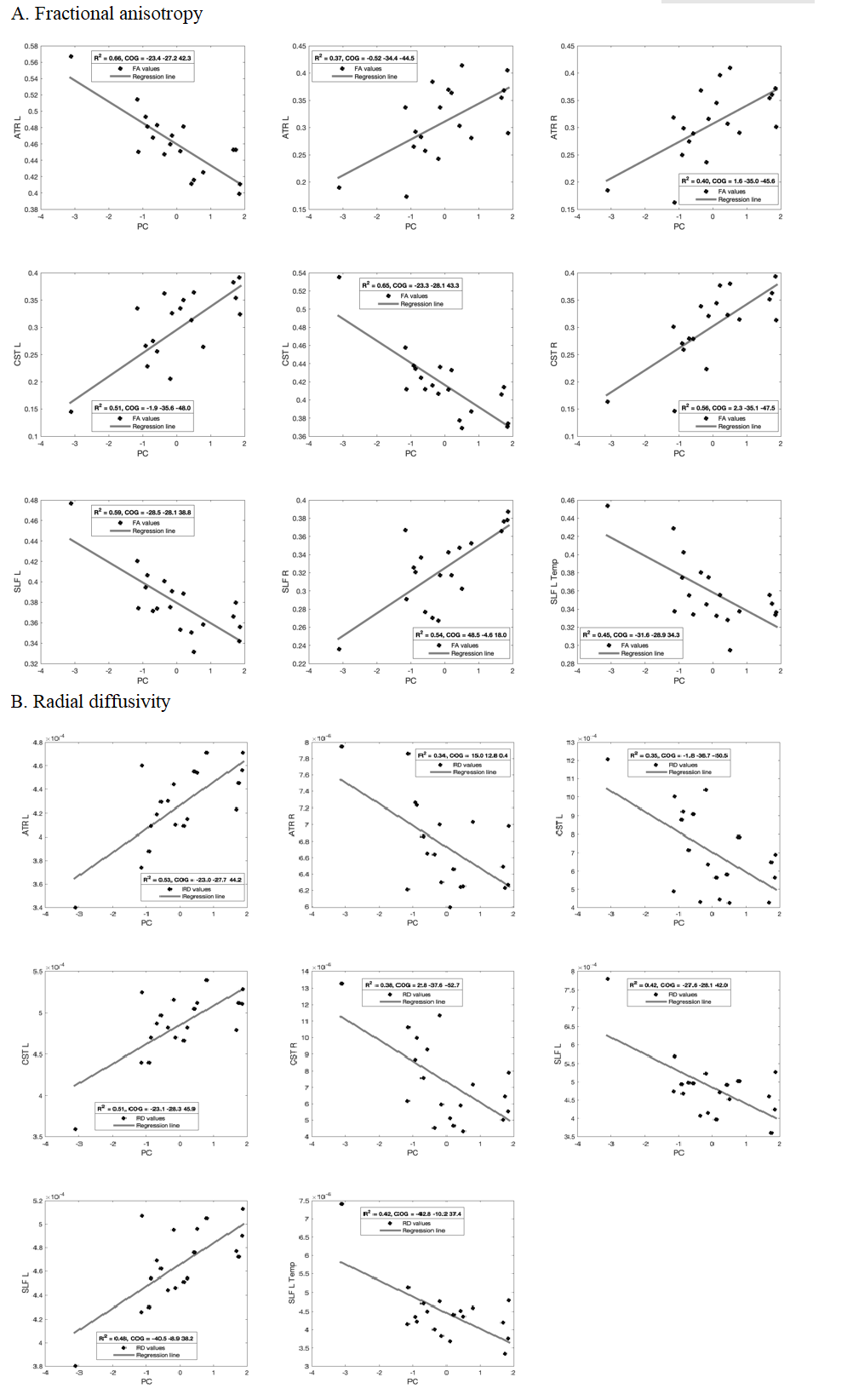

Supplement: S3 Fig — Fractional anisotropy (panel A) and radial diffusivity (panel B) values with respect to principal component values for each significant cluster. L = left. R = right. ATR = anterior thalamic radiation. CST = corticospinal tract. SLF = superior longitudinal fasciculus. COG = center of gravity. (TIF) [file pone.0274955.s003.tif]
